# Time resolved in-situ multi-contrast X-ray imaging of melting in metals

**DOI:** 10.1038/s41598-022-15501-2

**Published:** 2022-07-15

**Authors:** Lorenzo Massimi, Samuel J. Clark, Sebastian Marussi, Adam Doherty, Saurabh M. Shah, Joachim Schulz, Shashidhara Marathe, Christoph Rau, Marco Endrizzi, Peter D. Lee, Alessandro Olivo

**Affiliations:** 1 Department of Medical Physics and Biomedical Engineering, University College London, London, UK; 2 Department of Mechanical Engineering, University College London, Gower St, London, WC1E 6BT UK; 3 MicroWorks GmbH, Schnetzlerstraße 9, 76137 Karlsruhe, Germany; 4 Institute of Microstructure Technology, Karlsruhe Institute of Technology, 76021 Karlsruhe, Germany; 5 Diamond Light Source, Harwell Oxford Campus, OX11 0DE Didcot, UK; 6 Present Address: X-ray Science Division, Argonne National Laboratory, 9700 S Cass Ave, Lemont, IL USA

**Keywords:** Applied physics, Imaging techniques

## Abstract

In this work, the application of a time resolved multi-contrast beam tracking technique to the investigation of the melting and solidification process in metals is presented. The use of such a technique allows retrieval of three contrast channels, transmission, refraction and dark-field, with millisecond time resolution. We investigated different melting conditions to characterize, at a proof-of-concept level, the features visible in each of the contrast channels. We found that the phase contrast channel provides a superior visibility of the density variations, allowing the liquid metal pool to be clearly distinguished. Refraction and dark-field were found to highlight surface roughness formed during solidification. This work demonstrates that the availability of the additional contrast channels provided by multi-contrast X-ray imaging delivers additional information, also when imaging high atomic number specimens with a significant absorption.

## Introduction

X-ray multi-contrast imaging is based on the retrieval of the refraction and dark-field contrast channels in addition to conventional transmission. This is typically achieved by using optical elements such as phase or absorption gratings^1,2^. Usually, these elements need to be repositioned or translated with respect each other in order to probe the variation in the beam modulation induced by the sample. Therefore, the acquisition of multiple images is usually needed to retrieve the three contrast channels^3,4^. While single image approaches exist, these are limited in spatial resolution to the period of the used optical element. Here we exploit an alternative based on a dynamic implementation of beam tracking (BT)^5,6^. BT is a single mask phase imaging technique which works with both synchrotron and laboratory sources. Compared to other techniques such as grating interferometry and edge illumination, the use of a single mask greatly simplifies the experimental setup. The requirement of a single image to perform phase retrieval also allows combining the technique with a dynamic acquisition scheme based on the continuous translation of the mask. This provides time resolved images at the full spatial resolution, determined by the mask aperture size^7^. With the refinement of these phase imaging approaches, the applications of X-ray multi-contrast imaging are rapidly increasing, extending from the imaging of biological specimens to that of materials, with advantages especially in the investigation of materials with low X-ray absorption such as carbon plates or polymeric scaffolds^8–10^. In particular, dark-field demonstrated the ability to reveal features invisible in the others channels^11,12^. In addition, the higher contrast provided by phase over transmission combined with dynamic capability of BT, provided new insights in the imaging of melting metallic powders^6^,especially on the formation of molten tracks in laser additive manufacturing processes which were previously studied only with conventional transmission^13^. In the present work, we exploit the previously developed dynamic BT imaging technique to investigate, at a proof-of-concept level, the melting dynamics of a thick metallic slab with the main aim to understand if a multi-contrast approach can provide new qualitative and quantitative data compared to the conventional X-ray imaging. In particular, we imaged the melting of commercially pure titanium, for which X-ray attenuation is significant, by means of a high-power laser operated in air and argon atmospheres. We focused on the features visible under both conditions in each of the available contrast channels, finding in both cases that additional features are detected in both the phase and dark-field contrast channels. Thanks to its high sensitivity to density variations, the phase contrast channel allows to clearly distinguish the liquid phase (melting pool) from the solidified titanium, invisible in conventional transmission. Similarly, the dark-field combined with the standard deviation of refraction highlights surface unevenness originated by the melting/solidification processes. The improved visibility of new features have been exploited to perform quantitative analyses by means of image segmentation that can not be done using only conventional transmission. In addition, scanning electron microscopy (SEM) and micro computed tomography (micro-CT) have been used to identify the origin of the different features for which 2D imaging was not conclusive. The present work demonstrates that the availability of the phase and dark-field contrast channels provides additional information even when imaging specimens with significant absorption, offering new perspectives to the investigation of thermal processes in metals.

## Results

**Figure 1:**
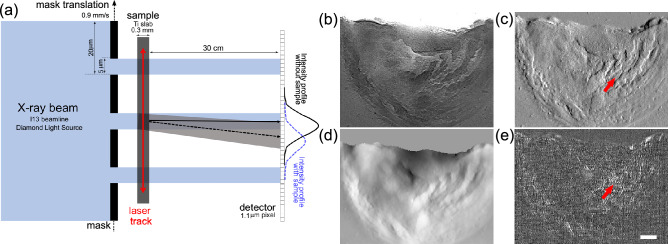
Panel (**a**) shows a schematic view of the experimental setup. Panels (**b**–**e**) show example transmission, refraction, phase and dark-field images of a melting titanium slab. In the phase image, the air region above the sample [panel (**d**)] has been set to zero by means of a mask obtained from the transmission image to reduce artifacts occurring from the integration of the refraction signal. Red arrows point at a highly scattering region. Scale bar is 200 μm for all images.

In the present work, an implementation of the beam tracking multi-modal X-ray imaging technique allowing for time resolved measurements is used to investigate the laser melting of a pure titanium slab in different atmospheres. A schematic view of the dynamic multi-modal acquisition setup is shown in Fig. 1, with an example of the different contrast channels obtained from the phase retrieval. The main beam is split into a series of beamlets by means of an absorption mask featuring a period of 20 μm and with 5 μm apertures. The latter defines the ultimate spatial resolution of the imaging system if an oversampling acquisition scheme, usually referred to as dithering, is used^7,14^. This consists of the acquisition of multiple images, which are subsequently digitally recombined, while translating the sample in sub-period steps to image all the parts of the sample that are otherwise covered by the mask septa. To make the dithering multi-image acquisition compatible with the requirement of time resolved imaging, we translated the mask continuously in front of the sample while the detector acquired a sequence of images. In the present experiment the maximum translation speed allowed by the used motor was 0.9 mm/s, which translates into a minimum exposure time of 5.6 ms per image. However, to achieve the final resolution equal to the aperture size of 5 μm, four sequential images were digitally recombined resulting in a final time resolution of 22.2 ms. A laser with variable power was used to melt a 300 μm thick titanium slab. While the laser moved back and forth [see panel (a)] over the slab’s upped edge, the beamlets’ shape was dynamically changed by the melting of the sample. The quantitative variation of the beamlets’ amplitude, centre and shape compared to the image without the sample provides the basis for the phase retrieval^3,5^. An example of the images obtained for each contrast channel while the laser-induced melting occurred in air are shown in Fig. 1b–e; note that the process outputs three images (b, c and e), and the phase image in (d) is obtained by integration of the refraction image in (c) (see “Methods”). A strong signal is detected in both transmission and phase with no significant differences between them, mainly due to the high thickness and density of the investigated material. In addition, many interfaces within the molten layer are formed as consequence of the interaction with oxygen, leading to strong signals in the refraction image. Conversely, a relatively low signal is detected only in some parts of the dark-field image [see red arrow in panel (e)]. This is due partly to the uniformity of the Ti slab, and partly to the fact that the relatively high achieved resolution (5 μm) allows to directly resolve most of the interfaces that form during the melting process, as illustrated by features pointed by red arrows in panels (c) and (e). In other words, all resolved interfaces are captured by the refraction image in (c), with only the neighbouring sub-resolution inhomogeneities ending up in the dark-field image in (e).

**Figure 2:**
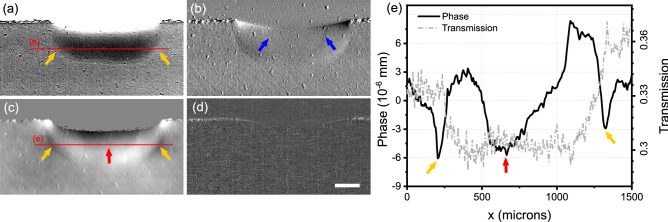
Panel (**a**–**d**) show the transmission, refraction, phase and dark-field contrast channels, respectively, of the Ti slab melting under fluxed Ar. The air region in the phase image [panel (**c**)] has been set to zero by means of a mask obtained from the transmission image to reduce artifacts occurring from the integration of differential phase signal. Blue arrows in panel (**b**) point at phase fringes originating from the edges of the liquid metal pool, which is indicated by the red arrow in panels (**c**) and (**e**). Yellow arrows in panels (**a**), (**c**) and (**e**) point at the edge of the re-solidified metal. Panel (**e**) shows line profiles extracted from the red lines shown in the transmission and phase images of panels (**a**) and (**c**). Scale bar is 200 μm for all images.

In order to investigate more in detail the different features visible during the melting process in each contrast channel, two different melting conditions have been imaged, initially with a constant laser power of 75W, namely melting under a fluxed argon atmosphere and in air. An example of the results obtained in the former case is shown in Fig. 2.

The transmission in panel (a) shows a homogeneous region of low transmission, formed while the laser moves over the surface (see Supplementary Video 1). The observed decreased transmission can be attributed to an increased amount of titanium along the beam path as a consequence of the melting process. However, refraction reveals the presence of a faint interface as shown by the blue arrows in panel (b), which highlights a density difference in the sample. This becomes more clearly visible in the integrated phase image [red arrow in panels (c)] and in the profile extracted from the phase image [solid line in panel (e)]. Note that because the sample is a slab and it is not fully contained into the field of view, the quantitative value of the integrated phase is not reliable and cannot be compared to the one expected for solid Ti. Such an interface is recognized as the actual interface between solid and liquid titanium (see also Supplementary Videos 2, 3 for the dynamic evolution of both refraction and phase contrast channels showing how this region follows the movement of the laser supporting its interpretation as the solid-liquid interface). In addition, a second faint layer is visible with a transmission slightly different from intact titanium which it is also well visible in the phase [see yellow arrows in panels (a) and (c)] and may be due to a different geometry of the solidified metal. In particular, it may be caused by solidification of liquid titanium that has bulged out since the laser may not be exactly centred on the thin upper surface of the titanium slab, while the power was sufficient to fully melt the entire thickness of the sample. It is worth noting that this interface, as well as the liquid pool interface, becomes progressively less visible in refraction as it becomes more horizontal (i.e. when approaching the bottom part), since the vertical orientation of the mask apertures yield phase sensitivity along one direction only. Remarkably, the line plots of Fig. 2e show that the solid-liquid interface is completely invisible in the conventional transmission image. Finally, no significant signal is present in the dark-field channel for the whole duration of the melting process (see Supplementary Video 4), revealing a homogeneous melting of the metal on a length scale below 5 μm.

**Figure 3:**
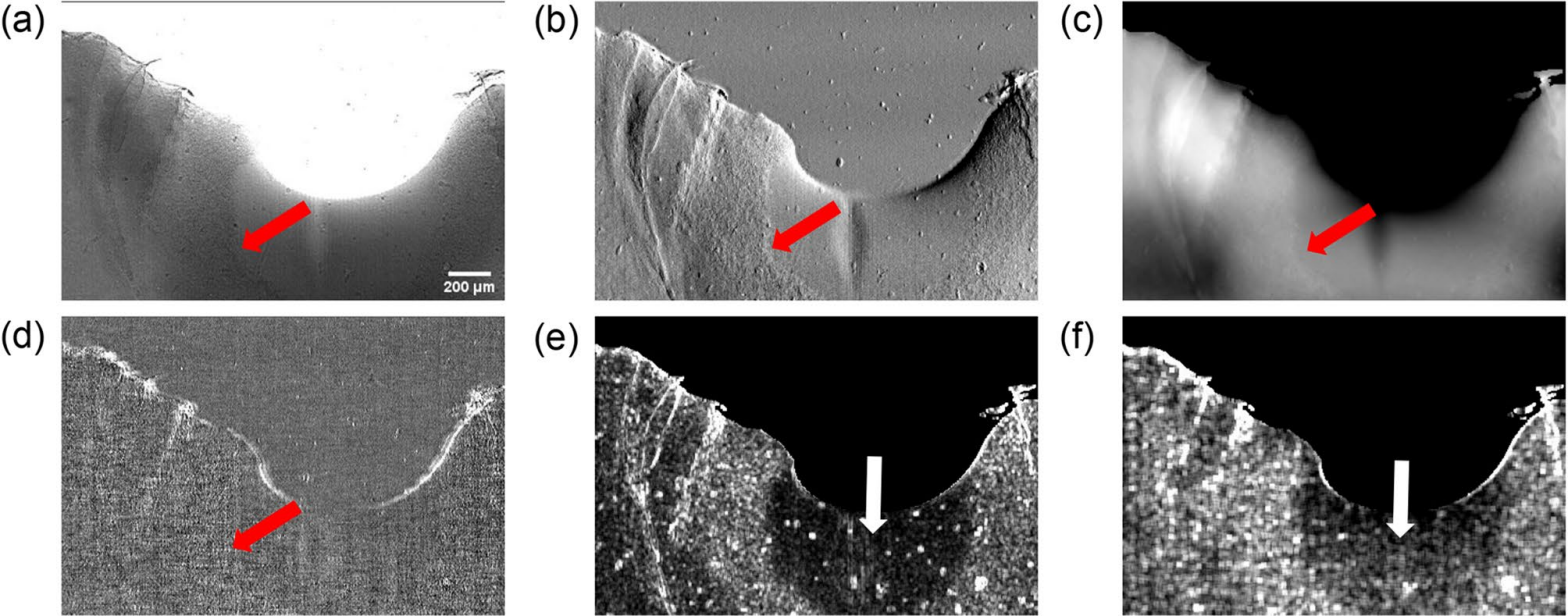
Panels (**a**–**d**) show the transmission, refraction, phase and dark-field contrast channels, respectively, for melting in air. For all the images the edge between oxidised layer and the molten pool is highlighted by red arrows. For both the refraction and dark-field images the image obtained by calculating the standard deviation of the signal on a sliding window of $3\times 3$, for the first and $5\times 5$ for the latter, is also reported in panels (**e**) and (**f**), respectively. Molten pool is indicated by white arrows in panels (**e**) and (**f**). Scale bar is 200 μm for all images.

In the second investigated case, the melting occurred in air leading to a more destructive process. In the presence of oxygen, the titanium alloy oxidised stabilising the hcp alpha-phase at first^15^. With further oxygen uptake various titanium oxides are expected to be formed; these create inhomogeneous structures compatible with the surface unevenness well visible in all the contrast channels, as shown in Fig. 3^15^. Also in this case, the formation of large pool is observed in the transmission and refraction channels of panels (a) and (b), respectively. The molten pool is, similarly to the case in argon, visible also as a region with lower density in the phase image shown in the panel (c). In addition, the solidified metal exhibits a clearly visible roughness which is visible in both channels and is compatible with the formation of titanium oxides, as previously mentioned^15^. However, the stronger signal at interfaces typical of refraction makes it more evident. This is further enhanced in the standard deviation of the refraction shown in panel (e), which also allows for an easier identification and segmentation of the interface of the molten pool indicated by white arrows. Similarly, such features appear in the scattering channel as a high frequency “salt and pepper” structure [red arrow in panel (d)]; we attribute this to the high spatial resolution achieved during the experiment, which means that only a minority of features with length scales below 5 micron end up in the dark field channel. Also in this case the use of the standard deviation better highlights the areas from which this signal originates, making the edge of the melted pool more clearly detectable as evident in the image in panel (f). The dynamic evolution of each contrast channel, i.e. transmission, refraction, phase and dark-field is reported in Supplementary Videos 5–8.

**Figure 4:**
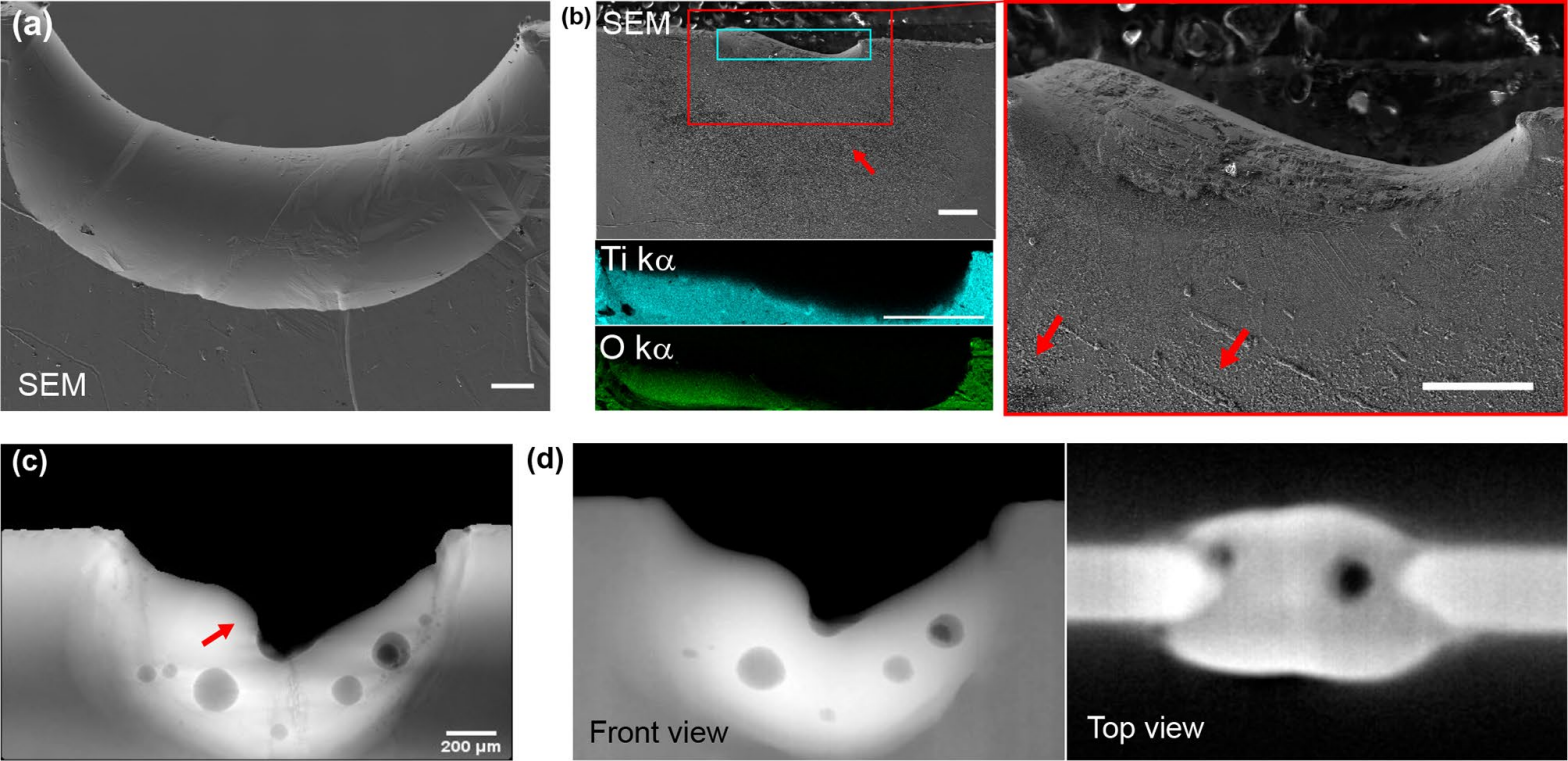
Panel (**a**) shows a SEM image of a Ti slab melted in argon flux, displaying a smooth appearance of the surface. Panel (**b**) shows a SEM image (left) and an image in the red rectangle acquired at higher magnification (right) of Ti slab partially melted in air. The EDS maps for the region in the light blue rectangle corresponding to the Ti-k$\alpha$ and O-k$\alpha$ are shown as insets below panel (**b**).The Red arrows point at surface roughness compatible with the creation of an oxide layer. Panel (**c**) shows an X-ray phase image of Ti molten in air. Panel (**d**) shows conventional micro-CT images of the same specimen seen from the front and from the top. Scale bar is 200 μm for all the images.

In order to support the interpretation, and in some cases investigate the origin of the different features observed in the different contrast channels, a multi-technique investigation has been carried out using a conventional micro-CT system as well as a scanning electron microscope (SEM). The results are reported in Fig. 4. SEM has been used to image two samples melted in Ar flux [panel (a)] and in air [panel (b)]. In the first case, as observed in the refraction and dark-field, no significant signal highlighting inhomogeneities is observed. This is supported by the smooth appearance of the surface in the SEM image. Conversely, as observed, melting in air leads to a rough surface producing a strong signal in the refraction image and in the dark-field. This roughness is clearly visible also in SEM images, see red arrows in panel (b) and relative magnified region, and is compatible with the oxidation of the surface in agreement also with previous qualitative findings^16^. In addition, EDS maps, shown as inset below panel (b), shows an homogeneous mix of both oxygen and Ti supporting the presence of an oxide layer. It is worth noting that the SEM images have been collected from samples where the laser melting had been interrupted after the initial steps, thus preventing the complete melting which occurred during the X-ray experiments. To investigate the origin of the higher density regions observed in the multi-contrast dynamic X-ray images, a micro-CT scan has been performed on an additional specimen that was also scanned during the synchrotron experiment. A region with brighter grey level is clearly visible in the phase image [red arrow in panel (c)] as well as in the corresponding micro-CT image shown in Fig. 4d. This is due to an increase in the thickness of Ti traversed by the beam, caused by the molten Ti dripping down along the slab and bulging out of it, as made evident by the top view of the micro-CT volume shown on the right of panel (d).

**Figure 5:**
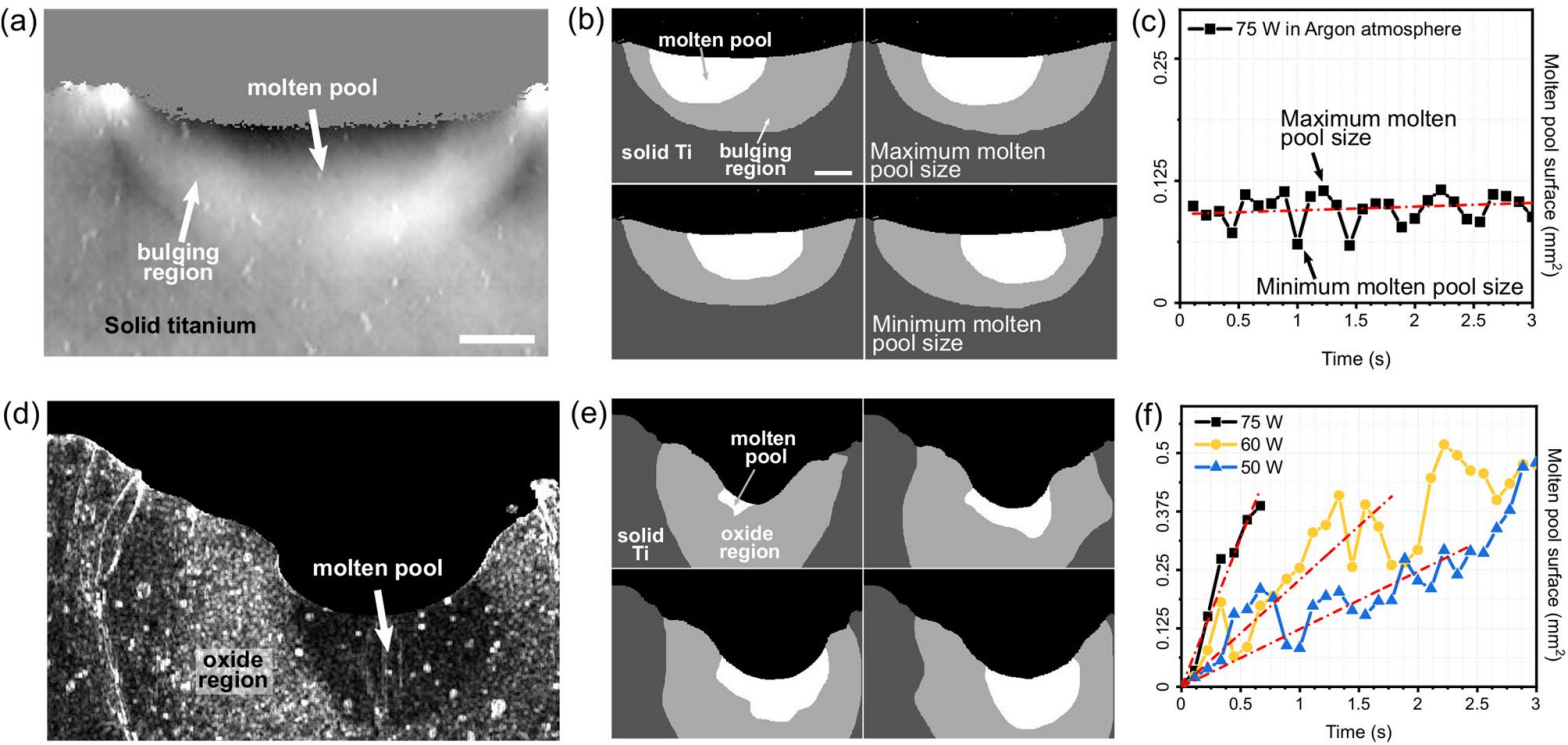
Panels (**b**) and (**c**) display the quantitative results extracted from the Ti slab melting under fluxed Ar, a representative phase image of which is shown in (**a**). Panel (**b**) shows exemplary results of the manual segmentation of the different regions i.e. melting pool, bulged region and solid Ti. Panel (**c**) shows the evolution of the cross section of the melting pool as a function of time, with a linear fit (dashed red line) superimposed to the experimental data. Similarly, panels (**d**) to (**f**) show the results of a similar analysis applied to the case where Ti is melted in air. Panel (**d**) shows an example of the standard deviation of the refraction signal, calculated on a $3\times 3$ kernel and used to segment the different regions reported in panel (**e**). Panel (**c**) shows the time evolution of the cross section of the melting pool for different laser powers. Scale bar for (**a**) and (**d**) is 200 μm. For all plots, the value $\Delta$t = 0 corresponds to the first image where the molten pool can be visually detected.

In both the investigated cases, i.e. Ti melting in argon and air, the different features observed during the experiment have been used to extract quantitative data by means of manual segmentation, as shown in Fig. 5. Specifically, when the Ti slab is melted in air, the improved contrast sensitivity demonstrated by X-ray phase contrast (see Figs. 2 and 5a) allowed segmenting the molten pool as well as the bulging region, as shown in Fig. 5b. Images segmented in this way can be used to extract quantitative parameters during the melting process, such as the evolution of the cross section of the molten pool as a function of time as reported in the graph in Fig. 5c. In particular, we found that the cross section of the molten pool is almost constant with very low increasing rate ($3.7 \times 10^{-6}$ mm^2^/s). It is worth noting that the molten pool becomes visible when it already has a large cross section, similar to its final value. This can be due to the fact that at the initial stage the molten pool is not large enough to produce a detectable contrast, to the time resolution achieved in this experiment being insufficient to capture this initial process, or to a combination of this factors. In addition, a periodic oscillation in the cross section can be observed from the graph, which can be explained by considering the back-and-forth movement of the laser beam during the experiment. In particular, a lower cross section of the molten pool is observed at the end of the track which may be due to the laser track not being exactly parallel to the edge of the Ti slab; this could bring the laser closer to the edge at the end of the track with more material bulging out of the pool and re-solidifying. When the Ti slab is melted in air, as already mentioned, the process is highly destructive. In this case, as shown in Fig. 3, the oxidation of the re-solidified surface introduces a surface roughness which provides a significant contrast against the liquid Ti pool when the standard deviation of the refraction signal is considered instead of phase, as shown in Figs. 3b and 5d. This contrast can be used to extract the molten pool as shown in Fig. 5e. Similarly, the oxidised region can be segmented, however, this is likely to have a lower accuracy since no sharp boundary with the solid Ti is observed. Also in this case, the segmented images can be used to calculate the growing rate of the cross section of the molten pool as shown in the graph in Fig. 5f for increasing laser powers. Remarkably, a decreasing linear growing rate is observed with decreasing powers, especially at the initial stage of the formation of the molten pool. Specifically, a linear fit of the values extracted from the initial part of the segmented frames reveals growing rates of $2.5 \times 10^{-2}$ mm^2^/s, $9.2 \times  10^{-3}$ mm^2^/s and $4.9 \times 10^{-3}$ mm^2^/s for laser powers of 75 W, 60 W and 50 W, respectively.

## Discussion

In this work the application of a recently developed dynamic beam tracking approach for time resolved multi-modal X-ray imaging is used to monitor the in-situ melting of a titanium slab^6^. Beam tracking has the advantaged to require a single absorption mask while two or three are commonly required. with other X-ray multi-contrast imaging techniques, such as grating interferometry or edge illumination^1,2^. The use of a single mask has a positive impact in terms of system stability, optimization and alignment. In addition, it can offer a cost-effective solution for the development of a commercial X-ray multi-contrast device. Beam tracking is also more versatile in terms of X-ray energies. Moreover, the possibility of using free standing absorption masks allows the use of low X-ray energies (<10 keV) which are required for imaging thin sections, which can be more complicated with optical elements requiring a substrate^17^. In conventional beam-tracking, the spatial resolution is determined by the size of the mask apertures if an over-sampled “dithered” acquisition is used^5,7^. To preserve this level of resolution in a dynamic imaging acquisition, the mask was translated continuously in front of the sample, with a speed corresponding to translating the mask by one aperture for each frame^6^. For the present experiment, this corresponded to a final time resolution of 22.2 ms due to a maximum motor speed of 0.9 mm/s. Despite the limitations of this proof-of-concept test, a number of features invisible to the conventional transmission channel have been observed, demonstrating the advantage of a multi-modal approach even when imaging samples with significant absorption such as metals, as previously proven on a different application^6^. Specifically, the phase channel provides a higher sensitivity to density changes, which enables a clear visualisation of the interface between solid and liquid metal invisible in the other channels. This is particularly evident in argon atmosphere where no destructive melting occurs. When Ti is molten in air the process is more destructive, and the molten pool becomes less evident (albeit still visible) also in the phase channel. However, the presence of oxygen leads to the formation of an oxide layer, producing a rough appearance of the surface which generates a strong signal in refraction and a less evident one in dark-field. In this case, the standard deviation of the refraction provides an additional tool to follow the evolution of the molten pool with time. By exploiting the phase and the standard deviation of refraction, it has been possible to follow the evolution of the molten pool with time. In particular, we observed that, when melting occurs in air, the size of the molten pool is almost constant over time, while it increases linearly with the time (at a rate depending on the laser power) when the melting occurs in air, especially at the beginning of the process. However, it is worth noting that the time resolution we achieved was two orders of magnitude below that commonly used for investigating additive manufacturing processes^13^. As a consequence, the initial stages of the formation of the molten pool may not have been resolved, leading to the constant pool size observed in the argon atmosphere. In addition, when fast changes occur, dithering recombination introduces a typical artifact characterized by a sawtooth shape of the edges, as visible in the videos. The achieved time resolution is not an intrinsic limit of the technique, as the bottleneck was caused by the limited speed of the mask translator: the use of faster translators may allow achieving a flux limited exposure time comparable to other dynamic melting and additive manufacturing experiments^13,18,19^. For example, with the same mask aspect ratio used for this experiment, a translation speed of 10 cm/s would allow to reach a final time resolution of 200 μs which is the same as reported in^20^. Interestingly, in both the investigated cases (melting in argon and air atmospheres), the dark-field presented a relatively weak signal, attributed to the homogeneous melting of the metal combined with the high spatial resolution of the system (5 μm), which enabled resolving most of the interfaces and material roughness directly in the refraction image. However, dark-field may still be useful in a flux-limited experiment where larger apertures may be required to improve the statistics per beamlet, reducing spatial resolution at the benefit of temporal resolution.

## Methods

### Melting system and sample

The melting system used for this work was a miniaturised scale custom additive manufacturing (AM) and laser materials processing process replicator, ISOPR^21^ . In this work, the 1070 nm $\hbox {TEM}_{{00}}$ laser was focused to a 50 μm spot and rastered back and forth along the top surface of commercially pure titanium substrates, 300 μm in thickness in the X-ray direction, with a power of 75 W and a scan speed of 1 mm/s. Laser melting was performed under a continuously fluxed argon atmosphere refreshed at a rate of 4 L/min or in air.

### SEM and micro-CT

The Ti weld samples were examined by scanning electron microscopy (SEM) in secondary electron imaging mode at 10 kV (Zeiss, Gemini), performing also energy-dispersive X-ray spectroscopy (EDS). The samples were also examined using a laboratory X-ray micro-computed tomography (μCT, Phoenix Nanotom, General Electric, USA). The voltage was set at 100 kV and beam current at 140 mA. 1000 projections were collected over 360°, with an exposure time of 1 s. The data were reconstructed using filtered back projection, resulting in a voxel resolution of 5.56 μm.

### X-ray data acquisition and processing

The experiment was performed at beamline I13 of the Diamond Light Source (UK), using a pink beam spectrum^22^. A 5 μm aperture size and 20 μm period absorption mask was placed 7 cm from the sample and used to implement beam tracking. The mask was manufactured by Microworks GmbH (Germany) by electroplating gold on a 200 μm thick silicon substrate. The detector was a PCO Dimax S4 coupled with a $\times 10$ optical lens, providing an effective pixel size of about 1.1 μm and a field of view of $2.2\times 2.2$ mm^2^. The detector was placed 30 cm downstream of the sample, which was the minimum distance allowed by the safety cabinet containing the melting rig. To achieve time resolved multi-modal images the mask was translated continuously in front of the sample with a speed of 0.9 mm/s, which was the maximum allowed by the used motor. This speed allowed a minimum exposure time of 5.6 ms per frame. After the experiment, the sample was manually removed, and a second acquisition was performed with the same parameters to obtain the flat fields. Laser triggered both the start of the mask movement and the camera acquisition. Each sequence of four subsequent frames, each acquired in a time corresponding to a lateral movement of the mask equal to an aperture and therefore covering an entire mask period, were recombined, resulting in a time resolution of about 22 ms and a spatial resolution of 5 μm. Due to the inaccurate return of the mask motor to its initial position after each sequence acquisition, a mismatch between flats and sample images was observed. Therefore, flat fields were acquired by collecting frames with the same exposure time of 5.6 ms but half the motor speed (0.45 mm/s), thus achieving a spatial oversampling of a factor of 2 compared to the acquisitions with the sample present. During the processing, the best matching flat field was automatically selected as the one with the maximum correlation with each of the sample images. Phase retrieval was performed by fitting a 5-Gaussian profile to line profiles obtained from each detector row over 5 adjacent beamlets, for images with and without the sample. In both cases, the key parameters (amplitude, centre and variance) were extracted from the fits for the 3 central Gaussians only, while the first and the fifth Gaussian were discarded to minimise the effect of cross-talk between neighbouring beamlets. The process was repeated on a rolling basis, ultimately extracting the above parameters for all beamlets both with and without the sample. The extracted amplitude, centre and variance values were then used to generate the transmission, differential phase and dark-field signals, respectively, by comparing values with and without the sample for each beamlet^5,23^. The process is performed over each individual detector row, then repeated over all image rows. Before phase retrieval, raw images were binned by a factor of 5 in the vertical direction, so as to approximately match the 5 μm resolution expected in the horizontal direction. Recombination of the dithering steps was performed for each contrast channel after phase retrieval. The obtained differential phase images were integrated to obtain the phase maps^24^. Phase images and standard deviation of refraction have been segmented manually to extract the size of the molten pool reported in the graphs. One image every five have been used for the segmentation to speed up the process.

## Supplementary Information

Supplementary Video 1.

Supplementary Video 2.

Supplementary Video 3.

Supplementary Video 4.

Supplementary Video 5.

Supplementary Video 6.

Supplementary Video 7.

Supplementary Video 8.

## Data Availability

The authors declare that the data supporting the findings of this study are available from the corresponding author on reasonable request.
